# Intersecting burdens: oral health, dietary patterns, food security and their impact on cardiometabolic risk and mental health in Ghana

**DOI:** 10.1186/s40795-025-01223-x

**Published:** 2025-12-19

**Authors:** Fiifi Amoako A. P. Essiam, Mary Amoako, Emmanuel Kwadwo Owusu, Evans Ogura, Eunice Akosua Serwaa Hammond, Ibrahim Ahmed Tijani, Fredrick Asenso Wireko, Lord Jephthah Joojo Gowans

**Affiliations:** 1 Department of Biochemistry and Biotechnology, Faculty of Biosciences, College of Science, Kwame Nkrumah University of Science and Technology, Kumasi, Ghana; 2 University Health Services, Kwame Nkrumah University of Science and Technology, Kumasi, Ghana; 3 Department of Mathematics, Kwame Nkrumah University of Science and Technology, Kumasi, Ghana; 4 School of Dentistry, Kwame Nkrumah University of Science and Technology, Kumasi, Ghana; 5 Cleft-Craniofacial Clinic, Komfo Anokye Teaching Hospital, Kumasi, Ghana

**Keywords:** Oral health, Food security, Dietary patterns, Mental health, Cardiometabolic health

## Abstract

**Background:**

The nutrition transition and persistent food insecurity in Ghana and similar low- and middle-income countries pose significant challenges to both physical and mental health. Emerging evidence suggests that oral health may be implicated in these associations. This study examines the associations between oral health, dietary patterns, food insecurity and their associations with cardiometabolic and psychological distress outcomes.

**Methods:**

This study employed a cross-sectional design, recruiting 100 participants in March 2025. Data on sociodemographic characteristics and dietary patterns were taken using standard questionnaires, while cardiometabolic risk factors were assessed using standard procedures. Mental Health was assessed using Kessler’s Psychological Distress scale, while food security was assessed using the USDA Household Food Security Questionnaire.

**Results:**

Four distinct dietary patterns were identified: Sweet Tooth, Traditional Ghanaian Staples, Animal-Based Protein, and Street Food. The Sweet Tooth pattern was significantly associated with poorer cardiometabolic health (β = 0.412, *p* = 0.031), while food insecurity showed a strong positive association with psychological distress (β = 0.594, *p* < 0.001). The Animal Protein Dominant Pattern was also linked to increased psychological distress (β = 0.174, *p* = 0.047). Oral health exhibited a marginal association with cardiometabolic health (β = -0.101, *p* = 0.075) but was not linked to psychological distress. Mediation analysis revealed that the relationship between food insecurity and psychological distress was partially mediated by increased consumption of street foods (β = 0.103, *p* = 0.014).

**Conclusion:**

This study highlights the detrimental impact of a sugary, energy-dense dietary pattern on cardiometabolic health and shows food insecurity as a key driver of psychological distress in Ghana. The partial mediation by street food consumption suggests that food environments contribute to mental health disparities among food-insecure populations. Public health interventions that use an integrated approach targeting food security, promotion of healthy diets and mental support are essential to address the dual burden of cardiometabolic and psychological disorder.

**Supplementary Information:**

The online version contains supplementary material available at 10.1186/s40795-025-01223-x.

## Introduction

Non-communicable diseases (NCDs), particularly cardiometabolic disorders such as hypertension and cardiovascular disease are escalating public health concerns in low- and middle-income countries undergoing rapid urbanization and lifestyle changes [1]. Ghana is a clear example of this trend, experiencing a dual burden of persistent undernutrition alongside rising rates of obesity and related chronic diseases [2]. These shifts are largely driven by changes in dietary behaviors and food security status which are further influenced by social determinants including oral health and mental well-being. In the quest to address the growing health challenges, understanding such complex interrelationships has become critical.

Food insecurity remains widespread in Ghana affecting an estimated 49.1% of Ghanaians, according to a nationally representative study [3] with higher prevalences in northern and rural regions. Defined as limited access to adequate and nutritious food, food insecurity not only compromises diet quality but also imposes a significant psychological toll [4, 5]. In 2014, Lachance, Sean Martin [5] noted that aside from affecting diet quality, food insecurity may impose a significant psychological toll on individuals. This assertion was supported by another research in 2024 [4] which reported that food insecurity was associated with higher levels of stress. This stress contributes to increased rates of psychological distress including anxiety and depression, which in turn can negatively affect health behaviors and outcomes. Food insecurity is also associated with overweight and cardiometabolic risk through mechanisms involving the consumption of inexpensive energy-dense but nutrient-poor foods [6]. Traditional diets, which are historically rich in tubers, cereals, legumes, fruits and fish now coexist with emerging westernized patterns that are characterized by high intakes of processed foods, refined sugars, saturated fats and animal proteins [7, 8]. This transition contributes to the increasing prevalence of overweight obesity and cardiometabolic risk factors observed in urban and peri urban populations.

Oral health, often overlooked but a pivotal component of our overall health, is also closely intertwined with nutrition [9] and systemic disease [10]. In Ghana, dental caries and periodontal disease affect over 50% of adults causing pain, infection and impaired nutritional intake [11]. Emerging evidence links oral health to systemic inflammation, which is long known to exacerbate metabolic risk factors such as hypertension and diabetes [12]. Moreover, oral health may influence dietary choices [13] and psychological well-being, while these factors in turn can affect oral health, creating a bidirectional relationship that complicates health outcomes. For instance, poor dentition may limit consumption of nutrient-rich foods like fruits, vegetables, and proteins, leading to nutritional deficiencies that may impair oral tissue repair and immune function. Simultaneously, psychological distress linked to oral distress may reduce self-care behaviors, while stress itself can exacerbate periodontal disease through inflammation pathways. Despite its importance, oral health remains underprioritized in public health policies and services in Ghana and most countries in sub-Saharan Africa [14].

Mental health disorders, including psychological distress, depression and anxiety, are increasingly recognized as major contributors to global health disease burden [15]. Globally anxiety and depression rank among the most prevalent mental health conditions. In Ghana, mental health services are limited, and stigma remains a significant barrier to care [16, 17]. Food insecurity has been robustly associated with psychological distress mediated by chronic stress and social vulnerability [18]. Additionally, poor diet quality and oral health problems may exacerbate mental health conditions, depicting the interconnectedness of physical and psychological health [19, 20]. In this study, psychological distress refers to non-specific symptoms of anxiety and depression, as measured by the Kessler Psychological Distress Scale (K10). The K10 is a validated screening tool that reflects general emotional strain and symptom severity rather than diagnostic categories of mental health [21].

Despite the multifaceted relationships between food security, dietary patterns, oral health, cardiometabolic risk, and mental health, research integrating these factors remains limited, especially in low- and middle-income countries. This study aims to address this critical gap by employing path analysis to clarify the direct and indirect pathways linking these variables, thereby providing comprehensive insights into their combined influence on health outcomes.

**Fig. 1:**
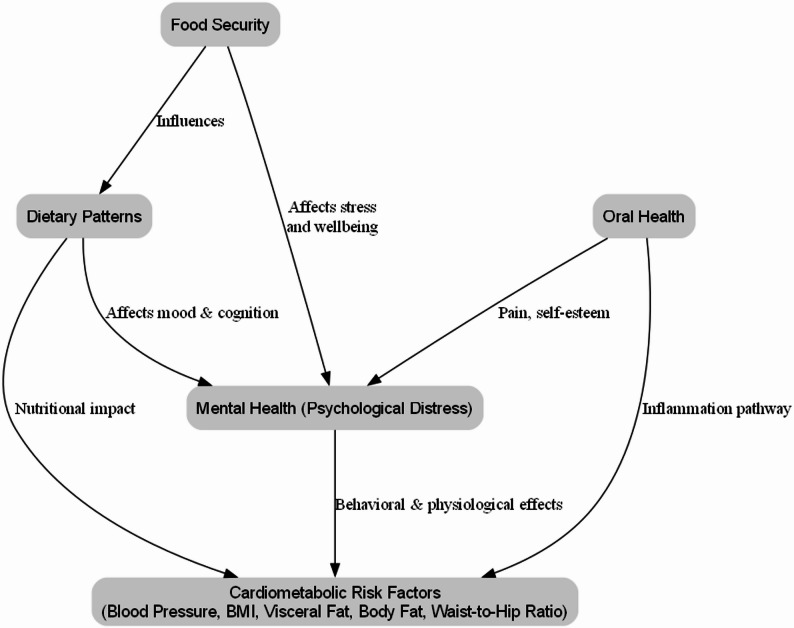
Conceptual framework of the study

Beyond Ghana and the sub-Saharan region, the findings of this research carry significant implications for global health. As many countries worldwide face similar nutrition transitions, rising food insecurity and increasing burdens of chronic diseases and mental health disorders, a holistic approach that addresses the interconnectedness of determinants of health is urgently needed. This study highlights the importance of integrated strategies that consider dietary quality, food access and oral health as critical components of efforts to reduce cardiometabolic and typological disease burden globally. Such evidence-based approaches can inform policies and programs aimed at fostering resilient healthier populations in diverse socioeconomic and cultural contexts. Figure 1 illustrates the hypothesized relationships among key study variables. Food security was posited to influence both dietary patterns and mental health. Dietary patterns and oral health were expected to exert direct and indirect influence on mental health and cardiometabolic risk with psychological distress serving as a potential mediator. This framework guided the selection of variables and the mediation analysis approach applied in this study.

## Methods

### Study design and site

This study employed a cross-sectional design. The study was conducted at the Dental Clinic at Kwame Nkrumah University of Science and Technology (KNUST) Hospital, Kumasi. KNUST Hospital is a secondary health facility that serves both the university community and the public, making it an appropriate setting for accessing a diverse adult population.

## Sampling and sample size

We follow the conventions of Jumbe, Comstock [22] to conduct an a priori power analysis. Assuming a conservative effect size ($\:{f}^{2}$ = 0.15), a two-tailed $\:\alpha\:$ of 0.05 and a $\:\beta\:$ of 0.05 (power = 95%), a sample size of 89 respondents was estimated to provide adequate power [22]. Assuming an anticipated non-response rate of 10%, the final sample size was adjusted to 100.

Participants were recruited using a convenience sampling technique, targeting adult outpatients attending the hospital during the study period. Eligible individuals were aged 18 years and above, provided informed consent, and were physically and mentally able to participate in the interview and clinical assessments.

## Study procedures

Data collection was conducted at the Dental Clinic of the KNUST Hospital between March and April 2025. All study procedures were performed by trained research assistants fluent in both English and Twi (the predominant local language in the Ashanti Region, Ghana). Questionnaires were administered through face-to-face interviews to ensure comprehension and consistency in responses. Participants were given the choice to respond in English or Twi, depending on their preference. The survey instruments, originally developed in English, were translated into Twi and backtranslated to ensure conceptual equivalence. No monetary incentives were provided, however participants were compensated for their time.

## Food security

Household food security was assessed using the USDA household food security survey module (HFSSM) which was previously used by [23], which captures experiences of food access and availability. The scale comprises items related to anxiety about food supply, food quality, and reductions in food intake or skipped meals due to financial constraints. Items were coded as 1 for often true and sometimes true, and 0 = never true. An individual who scored 0 was classified as food secure, 1–7 as moderately food insecure, and 8–15 as severely food insecure. The HFSSM has been used and evaluated in Ghana and other low and middle income countries [24, 25].

## Oral health

Oral Health was assessed using a structured questionnaire adapted from the World Health Organization’s Oral Health Survey methods, which has been previously used by Hou, Mi [26]. The instrument included self-reported indicators such as biting discomfort, toothache, bleeding gum, sleep disturbance due to oral pain, and social withdrawal related to oral issues. Responses were dichotomized and aggregated to classify participants as having either good oral health or poor oral health based on the presence or absence of multiple functional or physiological impairments. Participants who scored 22 or more were classified as having good oral health while those who scored below were classified as having poor oral health. This survey method has been previously used in Ghana by Hewlett, Blankson [14].

### Dietary patterns

Dietary assessment was done using a Food Frequency Questionnaire comprising of 60 commonly consumed food items in Ghana. The questionnaire’s food items were similar to those previously utilized by Abubakari and Jahn [27]. Participants were asked to recall how frequenctly they consumed each specific food item during the week before the assessment. Ratings ranged from 0 (indicating infrequent or no consumption of a particular food in the past week) to 6 (indicating consumption on more than six days in the past week). The food items were subgrouped into 14 groups for Principal Component Analysis (PCA) by aggregating scores of foods sharing similar returned characteristics while belonging to the same food groups.

## Anthropometric assessment and vitals

Anthropometric measurements were done following the standard procedures [28]. Weights and heights were measured using a bioelectric impedance analyser (OMRON, Model: HBF-514 C) and a stadiometer (Seca, Model: CE0123) respectively. Vital signs were also assessed using an automated blood pressure monitor (OMRON, Model: BP724N). Two readings were obtained at 1-minute intervals after the participant had been seated quietly for at least 5 min. These measurements were taken on the same day immediately after the interview.

## Mental health

Mental health was assessed using Kessler Psychological Distress Scale (K10), a validated 10 item instrument designed to measure symptoms of anxiety and depression experienced over the past four weeks [29]. Items include: “ During the last 30 days, about how often did you feel tired out for no good reason?”, ”During the last 30 days, about how often did you feel so sad that nothing could cheer you up?” and “How often do you feel worthless?”. Participants responded to each item on a 5-point Likert scale from 1 = None of the time to 5 = All of the time, with higher scores indicating greater psychological distress. For descriptive classifications, we used commonly reported cut offs (10–15 = no distress, 16–21 = mild distress, 22–29 = moderate distress and *≥* 30 severe distress) [30]. The K10 has shown good reliability and validity in Ghana and several other African studies [31–33].

### Data analysis

Data analysis was performed using R (version 4.5.1) and Python for Windows (version 3.12.7). Continuous variables were reported as mean and standard deviation, while categorical variables were presented as frequencies and percentages. Principal component analysis using varimax orthogonal rotation was carried out to identify dietary patterns from the food types. Factors with eigenvalues > 1 were retained and patterns were named based on factor loading > 0.5. The regression method was used to generate a factor score for each participant under each identified pattern. Structural Equation Modelling (SEM) was used to assess both Direct and indirect pathways among food insecurity, dietary patterns, oral health, cardiometabolic risk and mental health. A latent variable for cardiometabolic risk was constructed from five observed indicators: systolic blood pressure, diastolic blood pressure, body mass index, visceral fat and body fat percentage. Model estimation was conducted using the maximum likelihood estimator. Standardized coefficients were reported for ease of interpretation. Model fit was assessed using the comparative fit index and Tucker-Lewis Index. Although mediation analysis was conducted using SEM, the cross-sectional nature of the data precludes causal inference. Indirect effects should therefore be interpreted as statistical rather than causal mediation. Internal consistency (Cronbach’s α) for multi-item instruments was Kessler Psychological Distress Scale (α = 0.877), oral health questionnaire (α = 0.846) and USDA Food Security Questionnaire (α = 0.938).

## Results

### Socio-demographic characteristics of participants

The study comprised 100 participants, with a near-even gender distribution (53 males, 52.6%; 47 females, 47.4%). The participants was predominantly young, with 79% (*n* = 79) aged below 30 years, while only 2% (*n* = 2) were above 60 years. Age distributions were comparable between genders, with males and females showing similar proportions across all age categories (e.g., 77.36% vs. 80.85% for < 30 years; 1.89% vs. 2.13% for > 60 years). Educational attainment was high, with 83% (*n* = 83) having tertiary education, while only 3% (*n* = 3) reported no formal education or only basic education. Geographically, most participants resided in urban areas (77%, *n* = 77), with smaller proportions in peri-urban (9%, *n* = 9) and rural (14%, *n* = 14) localities. Urban residence was slightly more common among females (78.72%) than males (75.47%). This information is shown in Table 1.

**Table 1:** Sociodemographic characteristics of participants

Variable	Male (*n* = 53)	Female (*n* = 47)	Total (*N* = 100)
*Age*			
< 30	41 (77.36%)	38 (80.85%)	79 (79%)
31–40	3 (5.66%)	2 (4.26%)	5 (5%)
41–50	4 (7.55%)	3 (6.38%)	7 (7%)
51–60	4 (7.55%)	3 (6.38%)	7 (7%)
> 60	1 (1.89%)	1 (2.13%)	2 (2%)
*Marital Status*			
Single	44 (83.02%)	39 (82.98%)	83 (83%)
Married	9 (16.98%)	6 (12.77%)	15 (15%)
Widowed	0 (0%)	2 (4.26%)	2 (2%)
*Educational Level*			
None	0 (0%)	2 (4.26%)	2 (2%)
Basic Education	0 (0%)	1 (2.13%)	1 (1%)
GCSCE/SSCE/WASSCE	9 (16.98%)	5 (10.64%)	15 (14%)
Tertiary Education	44 (83.02%)	39 (82.98%)	83 (83%)
*Locality*			
Rural	7 (13.21%)	7 (14.89%)	14 (14%)
Peri-urban	6 (11.32%)	3 (6.38%)	9(9%)
Urban	40 (75.47)	37 (78.72)	77 (77%)

GCSCE/SSCE/WASSCE: General Certificate of Secondary Education/Senior Secondary Certificate Examination/West African Senior School Certificate Examination. Tertiary Education: Includes diplomas, undergraduate degrees, and higher qualifications

### Dietary patterns of participants

**Fig. 2:**
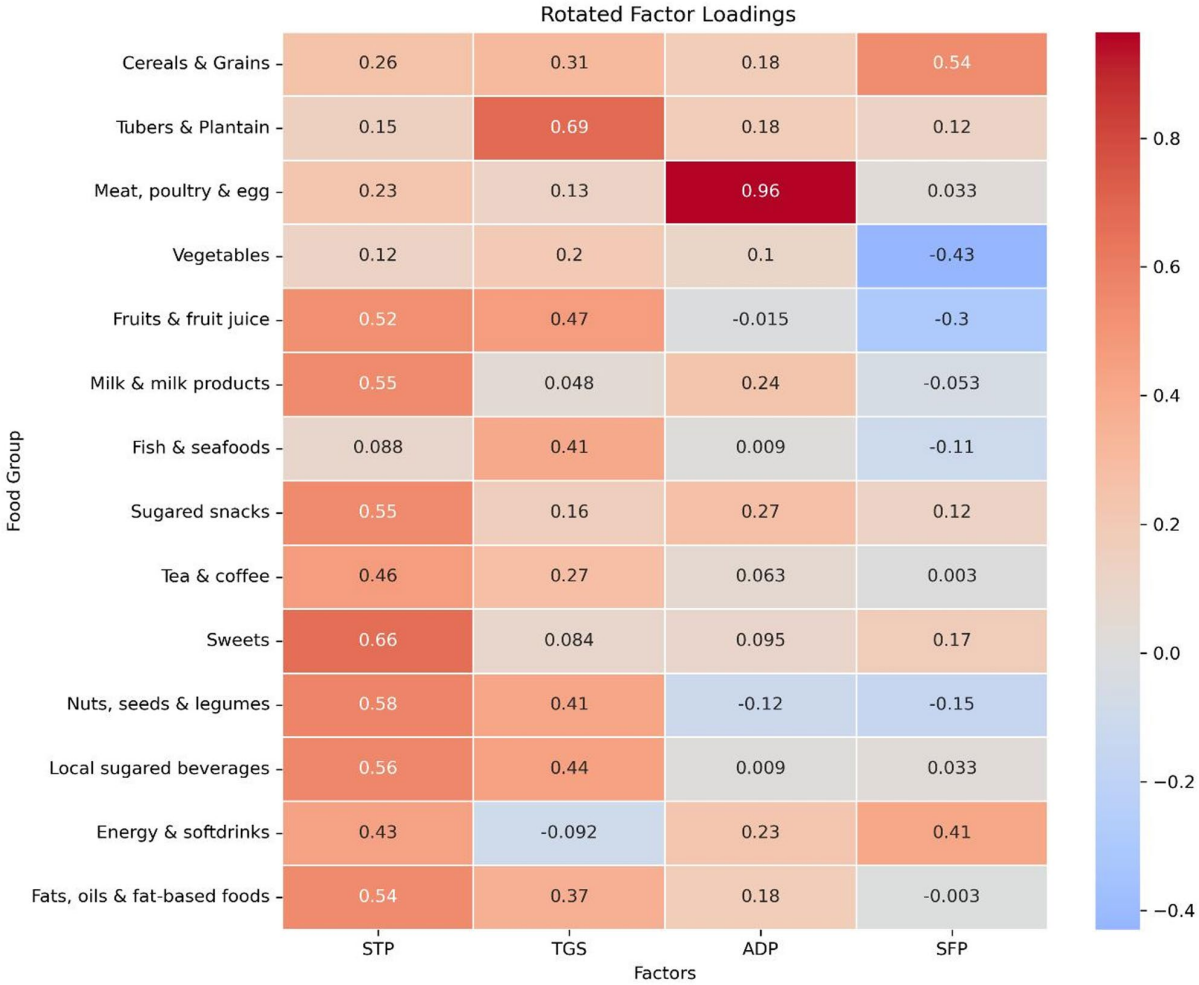
Heatmap of rotated factor loadings. Extraction method: principal component analysis. Rotation method: varimax with Kaiser normalization

Kaiser-Meyer-Olkin Measure of Sampling Adequacy (0.708) and Bartlett’s Test of Sphericity (χ^2^ = 413.41) showed that the data was adequate for PCA (*p* < 0.001). The principal component analysis identified four distinct dietary patterns based on participants’ food consumption habits (total variance = 60.18%). The first pattern, labelled the Sweet Tooth Pattern (STP), was characterized by high consumption of energy-dense and sugary foods, including sugared snacks, sweets, local sugared beverages, and fats/oils. The second pattern, termed the Traditional Ghanaian Staples (TGS), was dominated by traditional carbohydrate sources, particularly tubers and plantains, along with consumption of cereals/grains, legumes and nuts, fruits and fruit juices, milk and its products and fish/seafood. A third pattern, the Animal Protein Dominant Pattern (ADP) was distinguished by extremely high consumption of meat, poultry, and eggs. Lastly, the Street Food Pattern (SFP) was marked by positive loadings for cereals/grains, energy and soft drinks which typically reflects Ghanaian street foods such as kenkey, waakye, rice and beans. The heatmap in Fig. 2 shows the dietary patterns.

### Distribution of food security, oral health, and psychological distress among participants

**Fig. 3:**
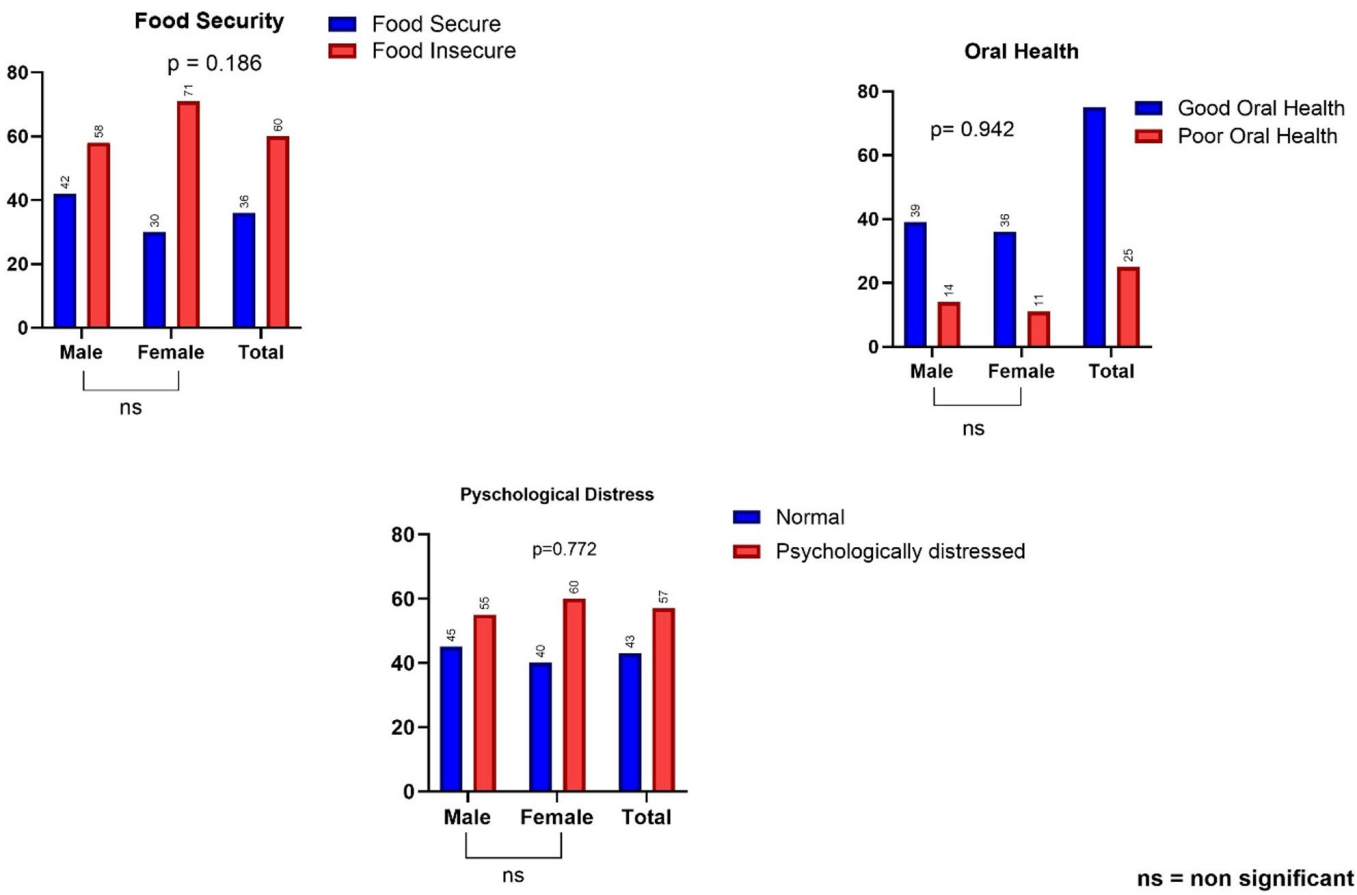
Distribution of Food Security, Oral Health, and Psychological Distress Among Participants. Food security status was classified using the USDA Scale [34]. Mild, moderate and severe food insecurity was classified as food insecure. Oral health was dichotomized as Good or Poor. Psychological distress levels were assessed via the K10 scale [35]. Mild, moderate and severe psychological distress was classified as psychologically distressed

Only 36.0% were food secure, while 44.0% experienced severe food insecurity and 20.0% reported moderate food insecurity. Oral health outcomes were relatively better, with 75.0% maintaining good oral health, though 25.0% had poor oral health. Psychological distress was prevalent, with 43.0% (*n* = 43) in the normal range, while 29.0%, 18.0%, and 10.0% reported mild, moderate, and severe distress, respectively. This information is shown in Fig. 3. The study also found hypertension, overweight/obesity and unhealthy visceral fat to be 21%, 43% and 10% respectively (shown in supplementary Table 1).

### Associations between dietary Patterns, psychosocial factors, and health outcomes

Table 2 shows the association between dietary patterns, psychosocial factors and health outcomes. The Sweet Tooth dietary pattern showed a significant positive association with poorer cardiometabolic health (β = 0.412, *p* = 0.031). Oral health exhibited a marginal trend towards cardiometabolic health (β = −0.101, *p* = 0.075). Food insecurity demonstrated a strong, significant positive relationship with psychological distress (β = 0.594, *p* < 0.001). The Animal-Based Protein pattern was also associated with distress (β = 0.174p = 0.047). Cardiometabolic health (β = 0.006, *p* = 0.497) and other dietary patterns (Sweet Tooth: β = −0.131; Traditional: β = −0.032; Street Food: β = −0.110; all *p* > 0.05) were not significant predictors. Oral health also showed no association (β = −0.027, *p* = 0.766).

**Table 2:** Direct effects of food Insecurity, dietary Patterns, and oral health on cardiometabolic and mental health

Predictor	Outcome	Std. Coefficient	Std. Error	*p*-value
Sweet Tooth Pattern	*Cardiometabolic health*	0.412	0.067	0.031*
Traditional Diet Pattern		0.040	0.075	0.240
Animal-Based Protein Pattern		0.067	0.066	0.791
Street Food Pattern		0.023	0.077	0.589
Food Insecurity		0.000	0.010	0.967
Oral Health		−0.101	0.014	0.075ϯ
Cardiometabolic health	*Psychological distress*	0.006	0.009	0.497
Sweet Tooth Pattern		−0.131	0.631	0.112
Traditional Diet Pattern		−0.032	0.816	0.728
Animal-Based Protein Pattern		−0.186	0.602	0.047*
Street Food Pattern		0.110	0.972	0.092
Food Insecurity		0.594	0.113	< 0.001**
Oral Health		−0.027	0.111	0.766

Adjusted for age and sex. Standardized coefficients (β) are reported. Significance levels: **p < 0.001. *p < 0.05, ϯ < 0.10, Cardiometabolic Risk was a latent variable constructed from five observed indicators: systolic blood pressure (SP), diastolic blood pressure (DBP), body mass index (BMI), visceral fat, and body fat percentage. Dietary patterns were derived from varimax orthogonal rotation from PCA

### Indirect effects of food insecurity via dietary patterns

**Fig. 4:**
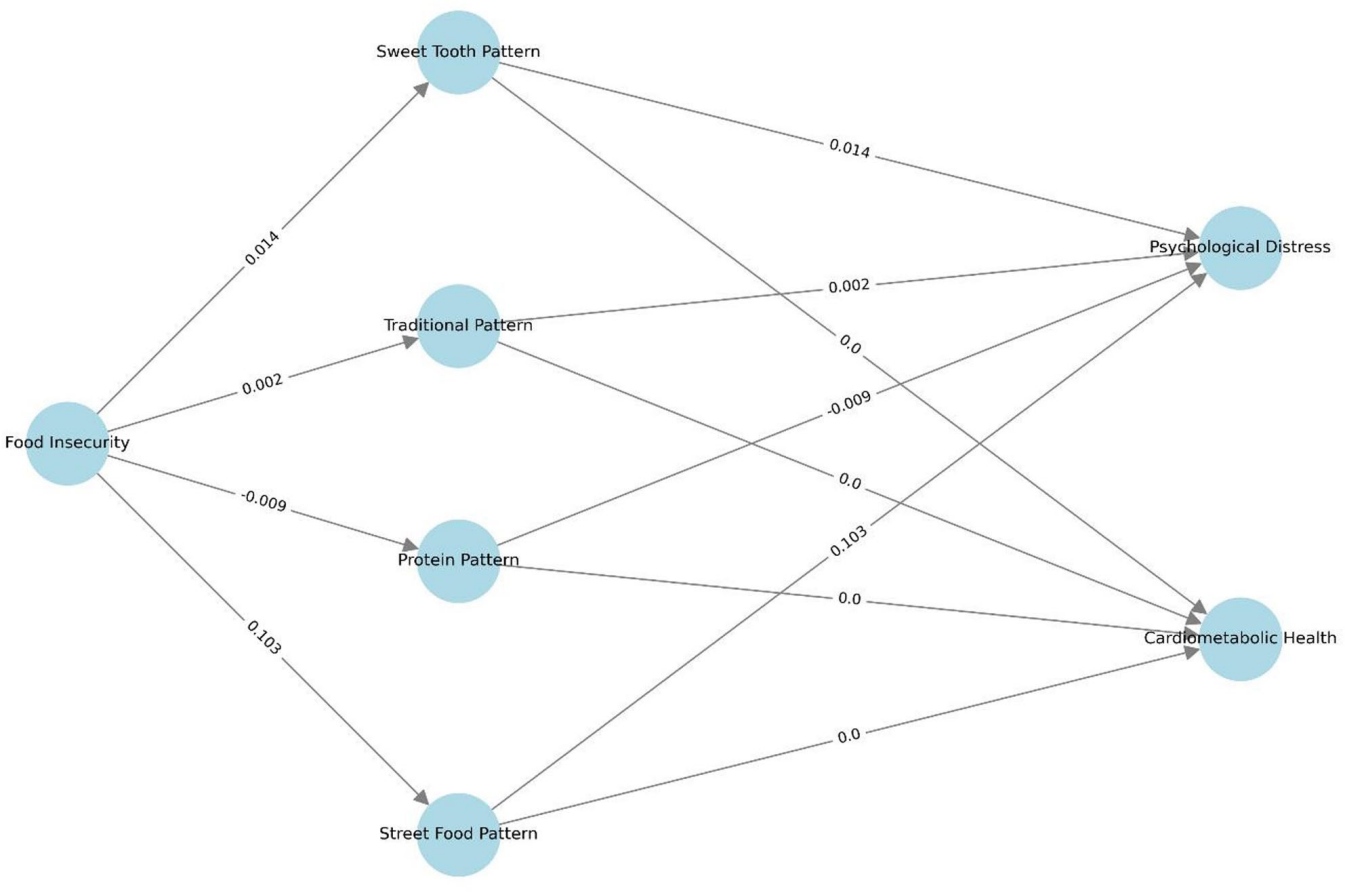
Structural Equation model results of observed relationships

Food insecurity showed a small but statistically significant indirect effect on psychological distress through increased consumption of street foods (β = 0.103, SE = 0.017, *p* = 0.014). No other dietary patterns demonstrated significant mediation effects for psychological distress, including sweet tooth (β = 0.014, SE = 0.022, *p* = 0.449), traditional (β = 0.302, SE = 0.015, *p* = 0.862), or animal-based protein patterns (β = −0.009, SE = 0.025, *p* = 0.678). For cardiometabolic health outcomes, none of the tested dietary patterns showed significant mediation effects between food insecurity and cardiometabolic health. The results are shown in Table 3. The pathway is shown in Fig. 4.

**Table 3:** Indirect effects of food insecurity on mental health and cardiometabolic health through dietary patterns

Mediation Path	Std. Indirect effect	Std. Error	*p*-value
Food Insecurity → Sweet Tooth Pattern → *Psychological distress*	0.014	0.022	0.449
Food Insecurity → Traditional Pattern → *Psychological distress*	0.002	0.015	0.862
Food Insecurity → Protein Pattern → *Psychological distress*	−0.009	0.025	0.678
Food Insecurity → Street Food Pattern → *Psychological distress*	0.103	0.017	0.014*
Food Insecurity → Sweet Tooth Pattern → *Cardiometabolic Health*	0.000	0.002	0.467
Food Insecurity → Traditional Pattern → *Cardiometabolic Health*	−0.001	0.002	0.679
Food Insecurity → Protein Pattern → *Cardiometabolic Health*	−0.001	0.002	0.916
Food Insecurity → Street Food Pattern → *Cardiometabolic Health*	0.000	0.001	0.874

Adjusted for age and sex. Standardized coefficients (β) are reported. Significance levels: **p < 0.001. *p < 0.05, ϯ < 0.10, Cardiometabolic Risk was a latent variable constructed from five observed indicators: systolic blood pressure (SP), diastolic blood pressure (DBP), body mass index (BMI), visceral fat, and body fat percentage. Dietary patterns were derived from varimax orthogonal rotation from PCA

## Discussion

This study sought to investigate the direct and indirect relationship between food security, oral health, dietary patterns and cardiometabolic and psychological well-being. The population showed high levels of food insecurity, psychological distress, hypertension and overweight/obesity and poor oral health. Four [4] distinct dietary patterns were identified, Sweet Tooth Pattern (STP), Traditional Ghanaian Staples (TGS), Animal-based Protein (ADP) and the Street Food Pattern (SFP). STP consumption was significantly associated with poorer cardiometabolic health, ADP and food insecurity were strongly associated with psychological distress. SFP was seen to mediate the association between food security and psychological distress.

### Food insecurity, psychological distress and oral health

The findings of this study reveal significant public health challenges in Ghana regarding food security and mental health particularly. Food insecurity emerges as a critical issue affecting over half of the population. Although our findings are higher than 2020 national estimates which report that only 11.7% [36] of Ghanaians are food insecure, they are similar to findings from Amoak, Braimah [37] who recently reported the prevalence of food insecurity in Ghana to be 64%. In the broader West African context, food insecurity has reached unprecedented level, with an estimated 27.3 million people facing acute food insecurity in 2022 and 40 million struggling during the 2024 post-harvest season. These figures indicate that the high rates observed in this study is consistent with most vulnerable sub-populations in Ghana and the wider West-African region. The drivers of this high food insecurity include high food prices and climate shocks [38] which are both relevant in Ghana.

Psychological distress in this study was also high (57%). A recent systematic review estimated that approximately 13% of adults in Ghana experience mental disorders although only a small fraction receive treatment [39]. Comparatively, studies among specific Ghanaian populations, such as studies among community-based mental health professionals, have reported clinically significant psychological distress rates ranging from 32.7% for stress to over 40% for anxiety [40]. Also, research among students (80%) and adolescents in Ghana indicates elevated levels of anxiety and depression [41, 42]. The persistent high prevalence of psychological distress in Ghana is compounded by systemic challenges including stigma around mental illness, low mental health literacy and inadequate healthcare infrastructure. Barriers such as mistrust and limited availability of mental health professionals hinder help-seeking behaviour exacerbating the burden of untreated psychological distress [43]. The Mental Health Act of 2012 in Ghana aims to improve access to care but implementation gaps remain and many individuals with distress remain under-served [44].

The prevalence of untreated oral diseases in Ghana ranges from 2.6% to 28.9% [11, 14]. This is similar to the 25% found in the present study. According to WHO’s 2022 oral health country profile for Ghana, untreated caries of permanent teeth affects approximately 25.3% of the population aged 5 years and older, while severe periodontal disease affects 29.3% of individuals aged 15 years and above [11]. Further supporting this, a population-based cross-sectional study conducted in Greater Accra Region found a high prevalence of missing teeth, retained roots and severe periodontitis areas [14]. Although Greater Accra, as Ghana’s capital city, has relatively better access to healthcare facilities, the persistence of oral health issues even in this urban setting suggests that factors beyond mere service availability may influence oral health outcomes, and socio-behavioural and cultural factors likely play a critical role in shaping oral health status. For instance, traditional oral hygiene practices such as the use of chewing sticks are widespread but may not be sufficient to prevent dental caries and periodontal diseases. These findings indicate that oral health is still a significant public health challenge in Ghana and hence reinforces the call for more comprehensive strategies that addresses both clinical and social determinants to reduce oral health disparities in Ghana.

### Dietary patterns of participants

Four [4] distinct dietary patterns were identified, Sweet Tooth Pattern (STP), Traditional Ghanaian Staples (TGS), Animal protein Dominated Pattern (ADP) and Street Food Pattern (SFP). These dietary patterns are consistent with findings from several Ghanaian studies that have similarly characterized dietary behaviours. A study conducted among adults in the Greater Accra region identified multiple dietary patterns Alo et al. [45], including a traditional pattern and a sweets and pastries pattern that closely resemble the TGS and STP found in the present study. The traditional pattern in that study was characterized by staple carbohydrates, legumes, fruits, and fish, mirroring the TGS’s emphasis on tubers, plantains, cereals, legumes, and fish/seafood. Likewise, the sweets and pastries pattern aligns with the STP’s focus on sugary snacks and energy-dense foods.

Among Ghanaian adolescents, a study in Northern Ghana identified two major dietary patterns: a sweet tooth pattern and a traditional pattern Abizari & Ali [7]. The sweet tooth pattern included sugar-sweetened snacks and beverages, similar to the STP in this study, while the traditional pattern was rich in cereals, local beverages, legumes, vegetables, and fish, paralleling the TGS. This similarity highlights the persistence of traditional dietary habits alongside increasing consumption of sugary and processed foods, reflecting the nutrition transition in Ghana. Further, research involving mother-child dyads in Ghana found dietary patterns labelled as beverage and sugary-based, meat-based, indigenous tuber-based, and indigenous grain-based diets Kubuga et al. [46]. The beverage and sugary-based pattern corresponds to the STP, while the meat-based pattern aligns with this study’s ADP, characterized by high meat, poultry, and egg consumption. The indigenous tuber- and grain-based patterns reflect the traditional staples pattern, rich in local carbohydrate-rich foods and legumes. These findings suggest that dietary patterns identified in this study are consistent with broader Ghanaian dietary behaviours across different population groups.

Nationally representative data from the Ghana Living Standards Survey also identified a traditional dietary pattern rich in starchy staples, fruits, vegetables, and fish, alongside a processed foods pattern including refined grains, processed meats, dairy, and sweets, and a food away from home pattern characterized by mixed dishes from restaurants Gersten et al. [8]. The traditional pattern corresponds to the TGS, while the processed foods and food away from home patterns share similarities with the STP and SFP, respectively. The identified dietary patterns are well aligned with existing Ghanaian research, showing common themes of coexistence between traditional diets rich in local staples and emerging patterns characterized by increased consumption of sugary, processed, and animal-source foods. The trends also indicate the rapid nutrition transition in Ghana.

### Sweet-Tooth pattern predicts cardiometabolic risk, while oral health shows marginal influence

The STP, characterized by high consumption of energy-dense, sugary foods and fats, showed a significant positive association with poorer cardiometabolic health. This finding aligns with extensive literature demonstrating that diets high in added sugars and unhealthy fats contribute to increased risks of obesity, insulin resistance, hypertension, and dyslipidaemia which are all key components of cardiometabolic syndrome [47, 48]. The STP’s strong predictive value for cardiometabolic health shows the detrimental impact of modern dietary shifts toward processed and sugary foods in Ghanaian populations undergoing nutrition transition.

The TGS was not significantly associated with cardiometabolic health or psychological distress suggesting that adherence to traditional diets rich in tubers, grains, legumes and fruits may neither exacerbate nor strongly protect against these health outcomes in this sample. However, the neutral findings may also reflect heterogeneity within traditional diets or insufficient power to detect subtle effects. The Street Food Pattern (SFP) did not also significantly predict cardiometabolic health (B = 0.023 *p* = 0.589) or psychological distress (B = 0.110, *p* = 0.092), although the positive trend towards psychological distress approached significance. Street foods often consist of refined grains fried snacks, and sugary beverages, which could contribute to poor health outcomes [49]. The lack of significance here may indicate variability in street food quality, quantity consumed or consumption patterns.

Oral health exhibited a marginal, non-significant trend toward association with cardiometabolic health (B = 0.001, *p* = 0.075) but no association with psychological distress (B = −0.027, *p* = 0.766). This may be partly explained by the relatively high proportion of participants reporting good oral health, limiting variability and subsequently constraining statistical power to detect significance. Although not statistically significant, the near-significant trend with cardiometabolic health aligns with emerging evidence linking poor oral health to systemic inflammation and cardiovascular risk [50]. This suggests a potential area for further research.

These findings suggest that reducing the consumption of energy-dense, sugary foods should be a central focus of cardiometabolic disease prevention in Ghana. Public health interventions could include taxation of sugar-sweetened beverages, community-based nutrition education and school or workplace initiatives promoting traditional diets rich in unrefined staples and locally available fruits and vegetables. Similar sugar-focused interventions in South Africa [51] and Mexico [52] have led to measurable declines in sugary beverage consumption and obesity rates, demonstrating potential relevance for Ghana’s policy context. Future studies should also incorporate objective oral health assessments such as dental charting and inflammatory biomarkers to more precisely quantify oral health and its systemic correlates.

### Food insecurity and animal-based protein predict psychological distress

Food insecurity demonstrated a strong and significant positive association with psychological distress, confirming well-established evidence that food insecurity is a critical social determinant of mental health [53]. The chronic stress, uncertainty, and deprivation associated with inadequate food access contribute to anxiety, depression, and other forms of psychological distress Myers [54], Shim & Compton [55]. This finding is particularly relevant in the Ghanaian context, where food insecurity remains a pressing issue.

Additionally, the Animal-Based Protein Pattern (ADP) was positively associated with psychological distress (B = 0.186, *p* = 0.037). This may reflect underlying socioeconomic or cultural factors. For example, higher consumption of animal protein could be linked to social pressures or economic stressors related to food affordability or availability, or it may indicate dietary changes associated with urbanization and lifestyle shifts that also influence mental health. Alternatively, it might reflect reverse causality or confounding factors not fully captured in the analysis. Further research is needed to elucidate the mechanisms behind this association. Specifically, research incorporating longitudinal or cohort designs to assess temporal relationships between dietary patterns and mental health outcomes would be phenomenal. Qualitative studies exploring individuals’ perception of animal-based food consumption, affordability and social meaning could provide contextual understanding of these associations. Integrating biomarkers of stress and inflammation and dietary intake data would help identify potential biological and behavioral mechanisms underlying this relationship.

The association between food insecurity and psychological distress highlights the mental health burden of inadequate food access. Social protection programs such as Ghana’s Livelihood Empowerment Against Poverty (LEAP) could integrate nutrition and psychosocial support components to buffer these effects. Also, food banks and community feeding programs may alleviate both hunger and chronic stress associated with food scarcity. The observed association between animal-based dietary patterns and psychological distress may reflect the stress of dietary and economic insecurity, suggesting the need for affordability-focussed interventions and nutritional counselling.

#### Street food consumption mediates the relationship between food insecurity and psychological distress

The mediation analysis revealed a statistically significant indirect effect of food insecurity on psychological distress through increased consumption of street food. This suggests that part of the adverse impact of food insecurity on mental health may be explained by greater reliance on street foods among food-insecure individuals. Street foods, often energy-dense but nutrient-poor, may provide affordable and accessible calories but lack essential nutrients, potentially exacerbating both physical and mental health problems [49]. This pathway highlights the importance of food environment and dietary quality in mediating the relationship between socioeconomic hardship and psychological outcomes.

No other dietary patterns significantly mediated the relationship between food insecurity and psychological distress, nor did any dietary patterns mediate the relationship between food insecurity and cardiometabolic health. This indicates that while diet quality and patterns are important, the direct psychosocial stressors of food insecurity may exert a more dominant influence on mental health than dietary factors alone.

The mediating role of street food consumption suggests that the local food environment may exacerbate the psychological toll of food insecurity. Urban food policies could therefore target improved regulation and health promotion among street food vendors, encouraging the inclusion of nutrient-rich options such as fruits, legumes and fortified foods.

### Strengths and limitations

This study has numerous strengths. It explores multiple interrelated factors such as dietary patterns, food insecurity, oral health, cardiometabolic risk and psychological distress, reflecting the real-world complexity of health determinants. Utilizing path analysis and identifying mediating relationships shows a higher level of analytical rigor in this study. There is also limited empirical data on how urban dietary transitions, food insecurity and mental health intersect in developing countries, filling a critical evidence gap. In LMIC settings linking oral health, cardiometabolic and mental health is novel hence, this study highlights psychological distress as a health burden associated with food security, hence advancing mental health nutrition data. This research is also relevant to SDGs 2 (zero hunger), SDG 3 (Good health and wellbeing), and SDG 10 (reduced inequalities) [56]. Despite these strengths, the study has some limitations that should be acknowledged. The cross-sectional design restricts the ability to infer causality or temporal direction between dietary patterns, food insecurity, and health outcomes, meaning that observed associations may be bidirectional or confounded by unmeasured factors. Dietary intake and oral health status were assessed through self-reported measures, which are vulnerable to recall bias and social desirability bias, potentially leading to misclassification or underreporting of unhealthy behaviours. Also, while the mediation analysis identifies statistical associations relevant to proposed pathways, these results should be interpreted as exploratory rather than confirmatory evidence of causal influence. Additionally, the study sample predominantly comprised educated, single, urban-based young adults which limits the generalizability of the findings to the wider Ghanaian population as food security experiences, dietary behaviours and oral health practices may differ substantially among rural, older and less educated groups. Oral health status was based on self-report rather than clinical examinations, which could introduce reporting bias. Finally, important confounding variables such as physical activity levels, healthcare access, and genetic predispositions were not included in the analysis, which may influence both dietary behaviours and health outcomes, potentially biasing the results.

## Conclusion

This study provides critical evidence on how dietary patterns, food insecurity, and oral health interact to influence cardiometabolic and psychological health outcomes. The identification of a Sweet Tooth dietary pattern significantly associated with poorer cardiometabolic health highlights the growing impact of nutrition transition, characterized by increased consumption of sugary and energy-dense processed foods. Food insecurity’s strong association with psychological distress emphasizes the profound mental health consequences of inadequate food access. Moreover, the partial mediation of food insecurity’s effect on mental health through increased street food consumption reveals how food environments shape health risks.These findings have broad public health relevance beyond Ghana. Across Africa and other low- and middle-income regions, rapid urbanization, globalization, and economic changes are driving similar shifts in dietary behaviours and food environments, contributing to the double burden of malnutrition-persistent undernutrition alongside rising obesity and noncommunicable diseases (NCDs). This study reinforces calls for integrated public health strategies that simultaneously address food insecurity, promote healthy traditional diets, regulate unhealthy food environments, and incorporate mental health support. Globally, as countries confront the escalating burden of diet-related chronic diseases, this research adds to the evidence base advocating for multisectoral policies targeting food systems, nutrition education, and social protection to improve population health sustainably. Mental health services should be embedded within nutrition and food security programs, recognizing that psychosocial distress and dietary inadequacy often co-occur.

## Supplementary Information

Supplementary Material 1.

## Data Availability

Data is available upon reasonable request from the corrresponding author.

## Abbreviations

NCDs: Non–communicable Diseases
SEM: Structural Equation Modelling
STP: Sweet Tooth Pattern
TGS: Traditional Ghanaian Staples
ADP: Animal–based Protein
SFP: Street Food Pattern
WHO: World Health Organization
USDA: United States Department of Agriculture
