# Supplementary material for: Intersecting burdens: oral health, dietary patterns, food security and their impact on cardiometabolic risk and mental health in Ghana
Source: BMC Nutr. 2025 Dec 19;11:228. doi: 10.1186/s40795-025-01223-x (PMC12750599; doi:10.1186/s40795-025-01223-x)
Supplement: Supplementary file 1 — Supplementary Material 1. [file 40795_2025_1223_MOESM1_ESM.docx]

Supplementary table 1. Prevalence of Food Insecurity, psychological distress and poor oral health among participants

| **Variable** | **Category** | **Frequency (n)** | **Percentage (%)** |
| --- | --- | --- | --- |
| **Food Security Status** | Food Secure | 36 | 36.0% |
|  | Moderate Food Insecurity | 20 | 20.0% |
|  | Severe Food Insecurity | 44 | 44.0% |
| **Oral Health Status** | Good Oral Health | 75 | 75.0% |
|  | Poor Oral Health | 25 | 25.0% |
| **Psychological Distress Level** | Normal | 43 | 43.0% |
|  | Mild Psychological Distress | 29 | 29.0% |
|  | Moderate Psychological Distress | 18 | 18.0% |
|  | Severe Psychological Distress | 10 | 10.0% |
